# The NSDHL/c-Myc/PKM2 Axis Drives Glycolytic Reprogramming and Tumor Growth in Multiple Myeloma

**DOI:** 10.3390/ijms27188239

**Published:** 2026-09-16

**Authors:** Can Yue, Bei-Hui Huang, Lin Qi, Xing-Ding Zhang, Juan Li

**Affiliations:** 1 Department of Hematology, The First Affiliated Hospital of Sun Yat-sen University, 58 Zhongshan Er Road, Guangzhou 510006, China; 18771798675@163.com (C.Y.);; 2 Shenzhen Key Laboratory for Systems Medicine in Inflammatory Diseases, Zhongshan School of Medicine, Shenzhen Campus of Sun Yat-sen University, 66 Gongchang Road, Shenzhen 518107, China

**Keywords:** multiple myeloma, NSDHL, glycolytic reprogramming, c-Myc, PKM2

## Abstract

Multiple myeloma (MM) remains largely incurable because of its complex biology and frequent disease relapse. Metabolic reprogramming is increasingly recognized as a critical contributor to MM progression. NAD (P)-dependent steroid dehydrogenase-like protein (NSDHL) has been highlighted as an unfavorable metabolism-related prognostic gene in plasma cell myeloma, but its functional significance and metabolic role in MM remain unclear. Publicly available MM transcriptomic datasets and the MMRF-CoMMpass cohort were analyzed to evaluate NSDHL expression and its clinical relevance. Cell proliferation was assessed using CCK-8 assays, while glucose consumption, lactate production, and extracellular acidification rate were measured to evaluate glycolytic activity. RNA sequencing was performed following NSDHL knockdown. NSDHL was upregulated in MM and associated with advanced disease stage and poor overall survival. NSDHL knockdown suppressed MM cell proliferation and glycolytic activity. Mechanistically, NSDHL reduced c-Myc phosphorylation at T58 while promoting phosphorylation at S62, thereby inhibiting proteasome-dependent degradation of c-Myc and stabilizing the protein. Stabilized c-Myc subsequently enhanced the transcriptional expression of Pyruvate kinase M2 (PKM2), leading to increased glycolysis. Our data reveal a novel NSDHL/c-Myc/PKM2 regulatory axis that drives glycolytic reprogramming and MM cell proliferation, highlighting NSDHL as a potential prognostic biomarker and therapeutic target in MM.

## 1. Introduction

Multiple myeloma (MM) is a malignant plasma cell disorder characterized by clonal expansion of plasma cells in the bone marrow and production of monoclonal immunoglobulins. Globally, approximately 188,000 new MM cases and 121,000 MM-related deaths were estimated in 2022, highlighting its substantial disease burden [1,2]. Despite major therapeutic advances, including proteasome inhibitors, immunomodulatory drugs, monoclonal antibodies and cellular immunotherapies, MM remains largely incurable because most patients eventually relapse or develop refractory disease [3,4]. Metabolic reprogramming is a key feature of cancer, allowing malignant cells to meet the energetic and biosynthetic demands required for sustained proliferation and tumor progression [5,6]. In line with this concept, metabolic reprogramming in MM cells is increasingly recognized as a key contributor to tumor growth, disease progression and therapeutic resistance [7]. Therefore, identifying key regulators of metabolic reprogramming may provide new mechanistic insight into MM progression and reveal potential therapeutic targets.

NAD (P)-dependent steroid dehydrogenase-like protein (NSDHL) is an enzyme involved in post-squalene cholesterol biosynthesis, where it participates in the C4-demethylation process during the conversion of lanosterol-derived sterol intermediates to cholesterol [8]. In addition to its established role in lipid homeostasis, recent studies have suggested that NSDHL may contribute to cancer progression, including breast cancer [9,10] and gastric cancer [11]. Mechanistically, NSDHL may promote malignant biological behaviors by regulating cholesterol biosynthesis, EGFR trafficking and degradation, TGFβ signaling [9,10,12]. Notably, NSDHL was included as an unfavorable component in a metabolism-related prognostic model for plasma cell myeloma, suggesting its potential clinical relevance in myeloma metabolism and prognosis [13]. Nevertheless, whether NSDHL has a functional role in MM progression, and whether it participates in metabolic regulation in MM, remains unclear.

Given the importance of metabolic reprogramming in MM and the emerging oncogenic role of NSDHL in other cancer types, we hypothesized that NSDHL may contribute to MM progression by regulating tumor cell metabolism. Therefore, this study aimed to elucidate the clinical relevance, biological function, and molecular mechanism underlying the role of NSDHL in MM. Elucidating NSDHL function in MM may uncover metabolic mechanisms of myeloma progression and therapeutic vulnerabilities.

## 2. Results

### 2.1. NSDHL Upregulation Was Associated with MM Progression and Promotes MM Cell Proliferation

**Figure 1:**
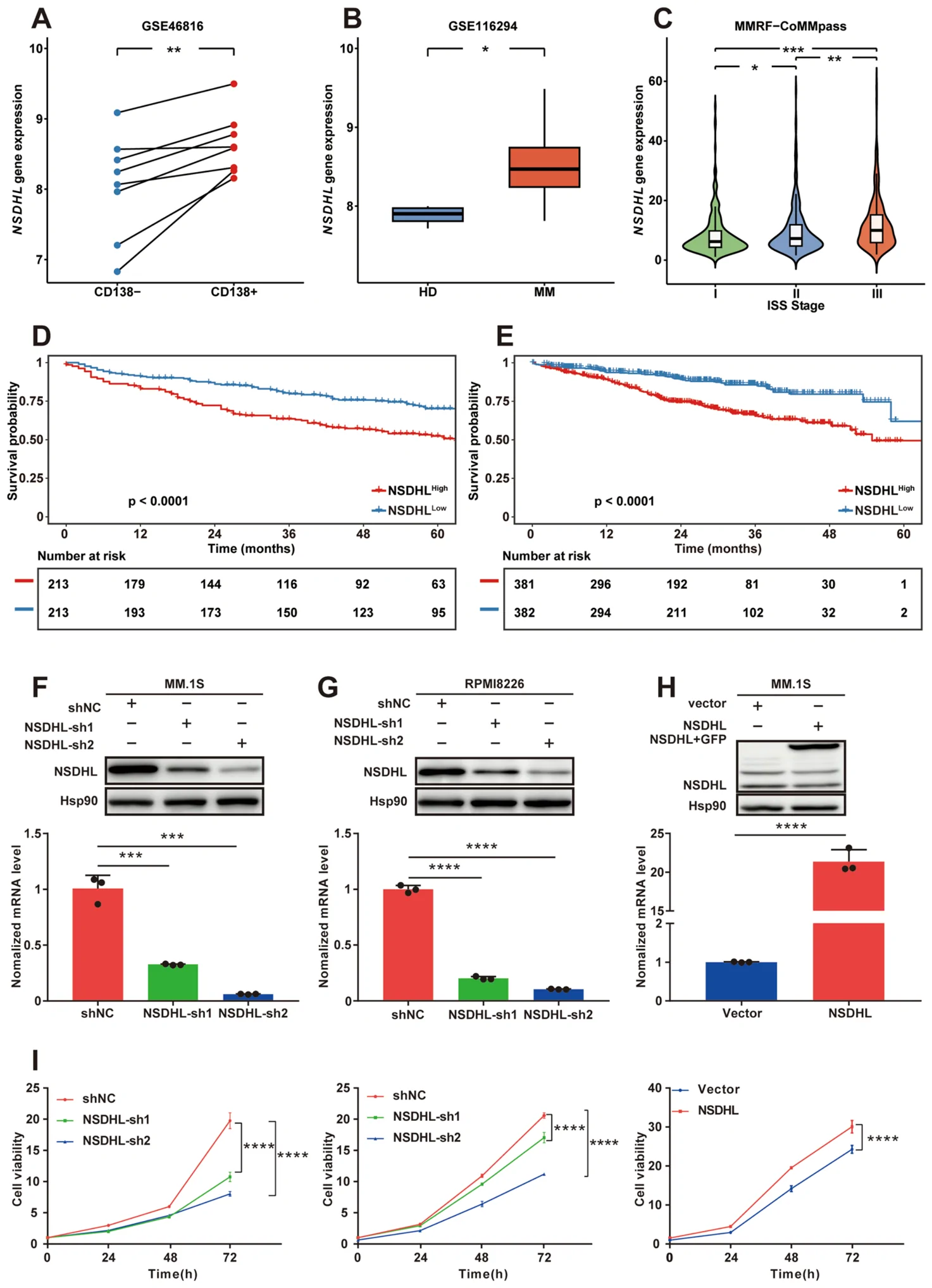
NSDHL was upregulated in MM and associated with disease progression and cell proliferation. (**A**) NSDHL expression in the GSE46816 dataset was compared between paired MACS-sorted CD138+ and CD138− fractions from 8 MM cell lines. (**B**) NSDHL expression in the GSE116294 dataset was analyzed in healthy donor (HD, *n* = 4) and newly diagnosed MM (NDMM, *n* = 50). (**C**) NSDHL expression was compared among patients with ISS stage in the MMRF-CoMMpass cohort, ISS stage I (*n* = 281), ISS stage II (*n* = 298), and ISS stage III (*n* = 258). (**D**,**E**) Kaplan–Meier survival analysis of overall survival in MM patients stratified by NSDHL expression in the GSE136337 dataset (**D**) and the MMRF-CoMMpass cohort (**E**). (**F**–**H**) qRT-PCR and Western blot analyses of NSDHL expression in MM.1S and RPMI8226 cells following NSDHL knockdown and in MM.1S cells with NSDHL overexpression. (**I**) CCK-8 analysis was performed to assess cell proliferation in vitro under the indicated conditions. Data are presented as mean ± SEM. * *p* < 0.05, ** *p* < 0.01, *** *p* < 0.001, **** *p* < 0.0001.

To determine the clinical relevance of NSDHL in MM, we first analyzed its expression in public datasets. In GSE46816, NSDHL showed higher expression in CD138^+^ cells than in CD138^−^ cells (Figure 1A), suggesting its potential involvement in malignant plasma cell biology. We then analyzed NSDHL expression in clinical samples using the GSE116294 dataset and found that NSDHL was significantly upregulated in newly diagnosed MM (NDMM) patients compared with healthy donors (HD) (Figure 1B). Moreover, analysis of the MMRF-CoMMpass cohort revealed that NSDHL expression increased progressively with advancing International Staging System (ISS) stage (Figure 1C). Kaplan–Meier survival analysis further showed that high NSDHL expression was associated with poorer overall survival in both the GSE136337 dataset and the MMRF-CoMMpass cohort (Figure 1D,E).

To further explore the biological role of NSDHL in MM cells, we knocked down NSDHL in MM.1S and RPMI8226 cells and overexpressed NSDHL in MM.1S cells. The efficiency of NSDHL knockdown and overexpression was confirmed by qRT-PCR and Western blotting (Figure 1F–H). Functionally, CCK-8 assays showed that NSDHL knockdown inhibited MM cell proliferation, whereas NSDHL overexpression promoted cell proliferation (Figure 1I). Taken together, these findings demonstrate that NSDHL is highly expressed in MM, correlates with disease progression and poor prognosis, and promotes MM cell proliferation.

### 2.2. NSDHL Promoted Glycolytic Reprogramming in MM Cells

**Figure 2:**
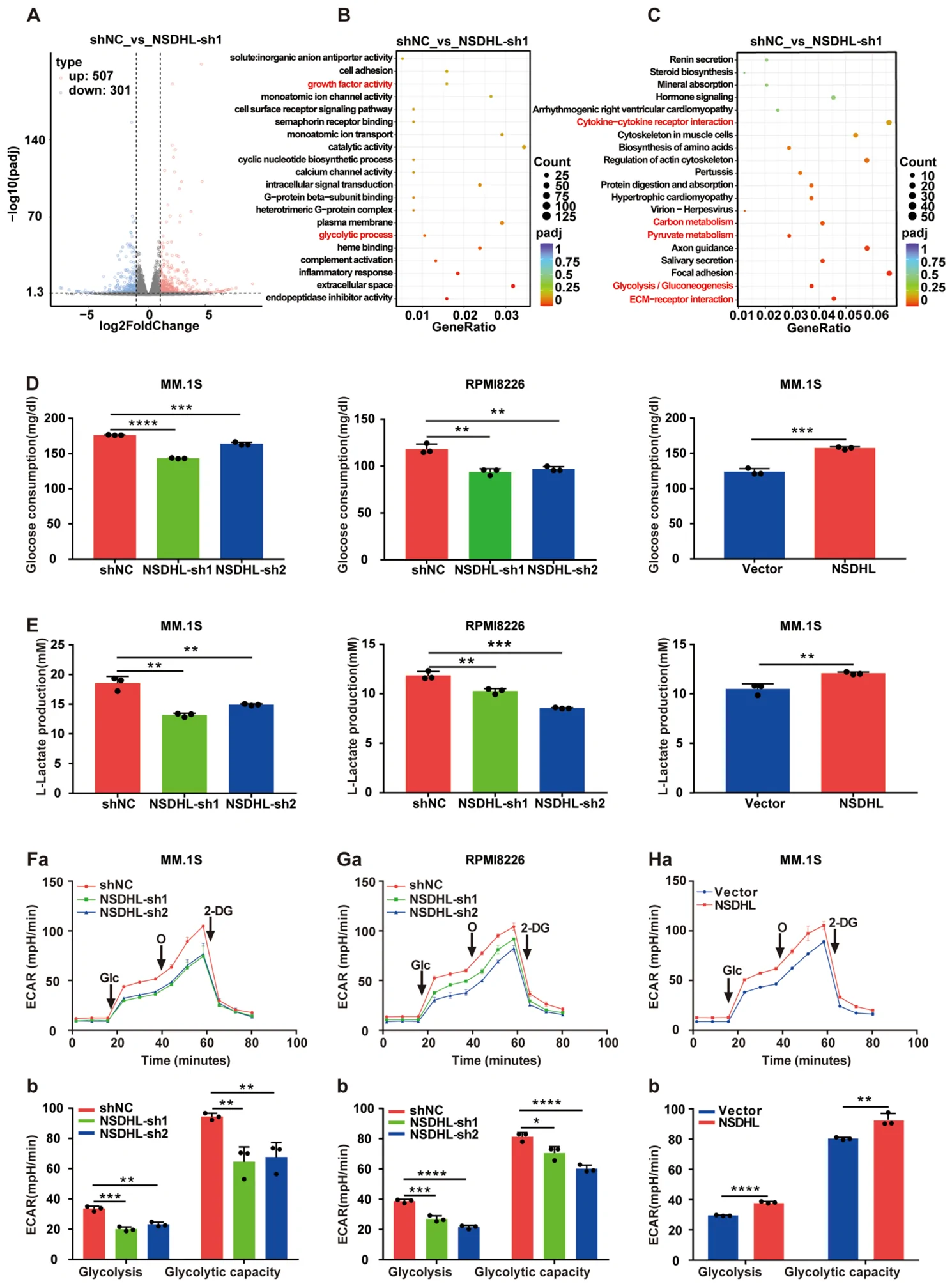
NSDHL promoted glycolytic reprogramming in MM cells. (**A**) Volcano plot showing differentially expressed genes identified by RNA-seq in MM.1S cells transduced with shNC or NSDHL-sh1. (**B**) GO enrichment analysis of NSDHL-regulated genes. (**C**) KEGG pathway enrichment analysis of NSDHL-regulated genes. (**D**,**E**) Glucose consumption (**D**), lactate production (**E**) were measured in MM.1S and RPMI8226 cells following NSDHL knockdown, as well as in MM.1S cells with NSDHL overexpression. (**F**–**H**) ECAR (**a**), glycolysis, and glycolytic capacity (**b**) were assessed in the indicated cells under the indicated conditions. Data are presented as mean ± SEM. * *p* < 0.05, ** *p* < 0.01, *** *p* < 0.001, **** *p* < 0.0001. ECAR, extracellular acidification rate; Glc, glucose; 2-DG, 2-deoxy-D-glucose; O, oligomycin.

To gain insight into the molecular pathways regulated by NSDHL in MM cells, we performed RNA-seq analysis in MM.1S cells transduced with shNC or NSDHL-sh1. Differential gene expression analysis revealed broad transcriptomic alterations following NSDHL knockdown, as shown by volcano plot (Figure 2A). To further characterize the biological significance of NSDHL-regulated genes, we performed Gene Ontology (GO) and Kyoto Encyclopedia of Genes and Genomes (KEGG) enrichment analyses. GO analysis revealed enrichment in terms related to growth factor activity and the glycolytic process (Figure 2B). KEGG pathway analysis further showed significant enrichment of cytokine–cytokine receptor interaction, carbon metabolism, pyruvate metabolism, glycolysis/gluconeogenesis, and ECM–receptor interaction pathways (Figure 2C).

Given the prominent enrichment of glycolysis-related metabolic pathways, we next examined whether NSDHL affects glycolytic activity in MM cells. Measurement of culture supernatants showed that NSDHL depletion markedly reduced glucose consumption and lactate production in both MM.1S and RPMI8226 cells, whereas NSDHL overexpression increased glucose consumption and lactate production in MM.1S cells compared with the empty vector control (Figure 2D,E). Consistently, ECAR profiling further confirmed these metabolic changes, showing that NSDHL knockdown attenuated basal glycolytic activity and glycolytic capacity, while ectopic NSDHL expression enhanced the glycolytic phenotype (Figure 2F–H). Collectively, these findings indicate that NSDHL promotes glycolytic reprogramming in MM cells.

### 2.3. NSDHL Promoted MM Cell Proliferation by Enhancing Glycolysis

**Figure 3:**
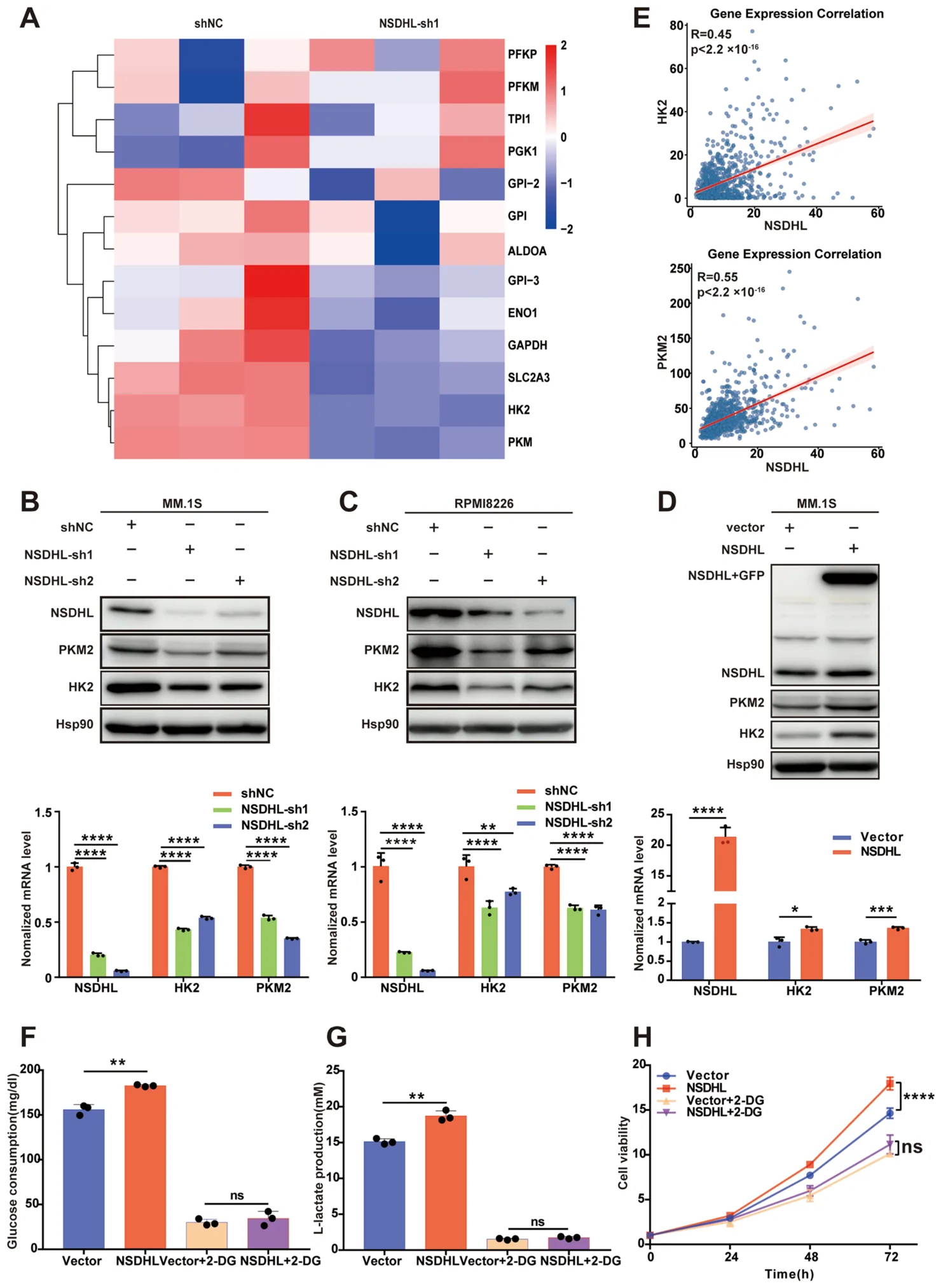
NSDHL promoted glycolysis-dependent proliferation and upregulated glycolytic enzymes in MM cells. (**A**) Heatmap of glycolysis-related genes expression identified by RNA-seq in MM.1S cells transduced with shNC or NSDHL-sh1. (**B**–**D**) qRT-PCR and Western blot analyses of HK2 and PKM2 expression in MM.1S and RPMI8226 cells following NSDHL knockdown, as well as in MM.1S cells with NSDHL overexpression. (**E**) Spearman correlation analysis of NSDHL expression with HK2 and PKM2 expression in MM patient samples from the GSE26760 dataset. (**F**,**G**) Glucose consumption (**F**) and lactate production (**G**) were measured in MM.1S cells following NSDHL overexpression with or without 2-DG treatment (10 mM). (**H**) CCK-8 analysis was performed to evaluate cell proliferation under the indicated conditions. Data are presented as mean ± SEM.* *p* < 0.05, ** *p* < 0.01,*** *p* < 0.001,**** *p* < 0.0001; ns, not significant.

Next, we sought to identify the mechanism by which NSDHL regulates glycolytic metabolism in MM cells. We therefore analyzed glycolysis-related genes from the RNA-seq data and found that multiple glycolytic regulators were altered following NSDHL knockdown in MM.1S cells, as shown by heatmap analysis (Figure 3A). Among these genes, Hexokinase 2(HK2) and Pyruvate kinase M2 (PKM2), two key enzymes involved in glycolytic flux, were markedly downregulated upon NSDHL depletion. Consistently, qRT-PCR and Western blot analyses demonstrated that NSDHL knockdown reduced both the mRNA and protein levels of HK2 and PKM2 in MM.1S and RPMI8226 cells, whereas ectopic NSDHL expression increased their expression in MM.1S cells (Figure 3B–D). To further support these findings, we analyzed MM patient samples from the GSE26760 dataset and found that NSDHL expression was positively correlated with HK2 and PKM2 expression (Figure 3E).

Based on the stimulatory effect of NSDHL on glycolytic activity, we next investigated whether enhanced glycolysis contributes to NSDHL-driven MM cell proliferation. Treatment with the glycolytic inhibitor 2-DG markedly suppressed the increases in glucose consumption and lactate production induced by NSDHL overexpression (Figure 3F–G). Moreover, 2-DG largely abolished the proliferative advantage conferred by NSDHL overexpression, as determined by CCK-8 analysis (Figure 3H). Together, these results suggest that NSDHL promotes MM cell proliferation, at least in part, by enhancing glycolytic metabolism.

### 2.4. NSDHL Positively Regulated c-Myc in MM Cells

**Figure 4:**
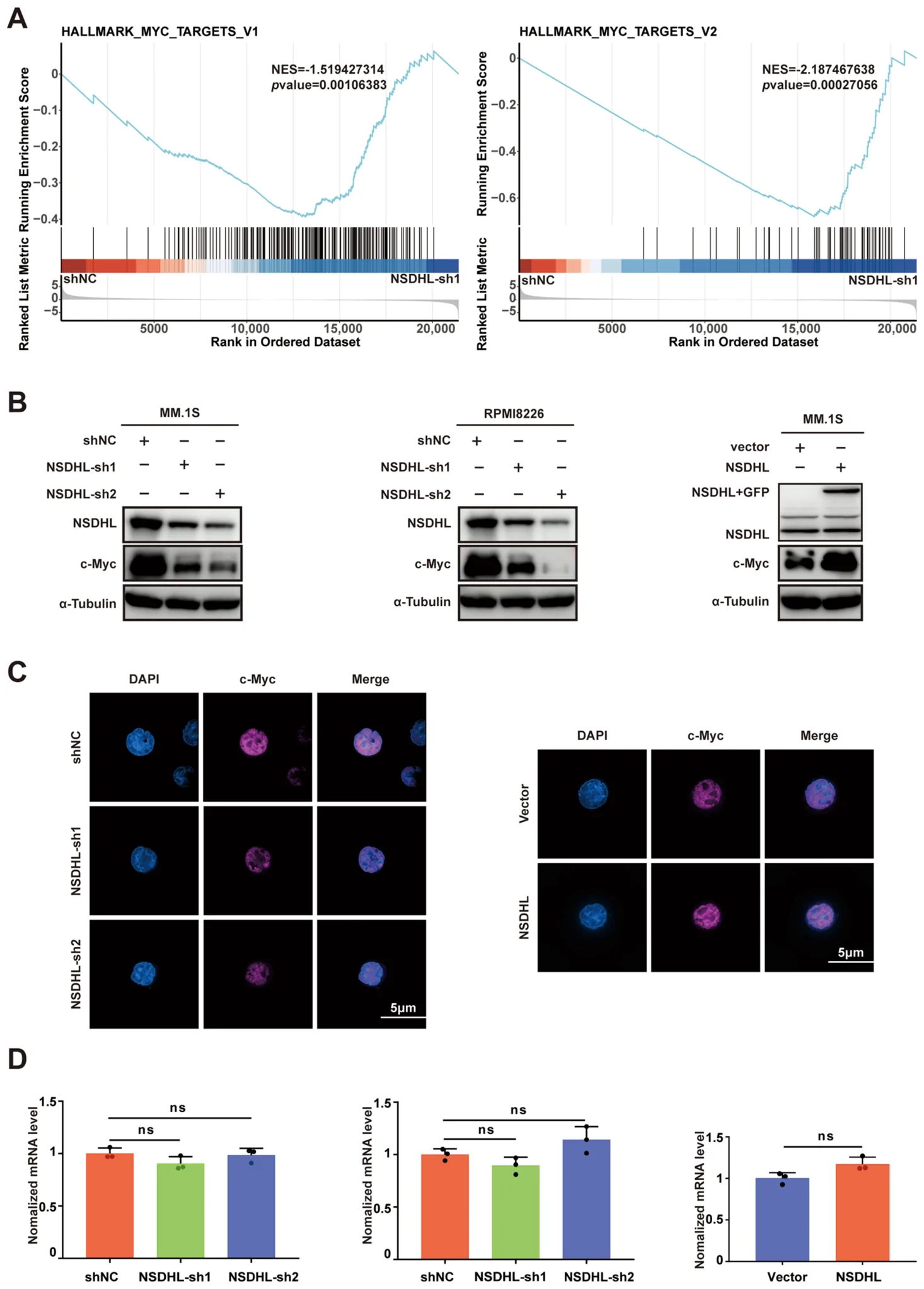
NSDHL positively regulated c-Myc in MM cells. (**A**) GSEA of RNA-seq data for MYC target gene signatures in NSDHL-sh1 MM.1S cells compared with shNC cells. (**B**) Western blot assay was performed to examine c-Myc expression in MM.1S and RPMI8226 cells with NSDHL knockdown, and in MM.1S cells with NSDHL overexpression. (**C**) Immunofluorescence staining was conducted to visualize c-Myc expression in MM.1S cells with NSDHL knockdown or overexpression. (**D**) qRT-PCR analysis of c-Myc mRNA expression in the indicated cells. Data are presented as mean ± SEM. ns, not significant.

To further explore the downstream mechanism by which NSDHL promotes glycolysis and proliferation in MM cells, we performed Gene set enrichment analysis (GSEA) of RNA-seq data. The results showed that MYC target gene signatures were significantly suppressed in NSDHL-sh1 cells compared with shNC cells (Figure 4A). We therefore examined whether NSDHL regulates c-Myc expression in MM cells. Western blot analysis showed that NSDHL knockdown markedly reduced c-Myc protein levels in MM.1S and RPMI8226 cells, whereas NSDHL overexpression increased c-Myc protein expression in MM.1S cells (Figure 4B). This finding was further supported by immunofluorescence staining, which showed decreased c-Myc expression following NSDHL knockdown and increased c-Myc expression upon NSDHL overexpression in MM.1S cells (Figure 4C). In contrast, qRT-PCR analysis showed no significant change in c-Myc mRNA expression under these conditions (Figure 4D), suggesting that NSDHL may regulate c-Myc primarily at the protein level rather than at the transcriptional level. Together, these findings suggest that NSDHL promotes c-Myc protein expression as a downstream effector in MM cells.

### 2.5. NSDHL Stabilized c-Myc by Suppressing Proteasome-Dependent Degradation in MM Cells

**Figure 5:**
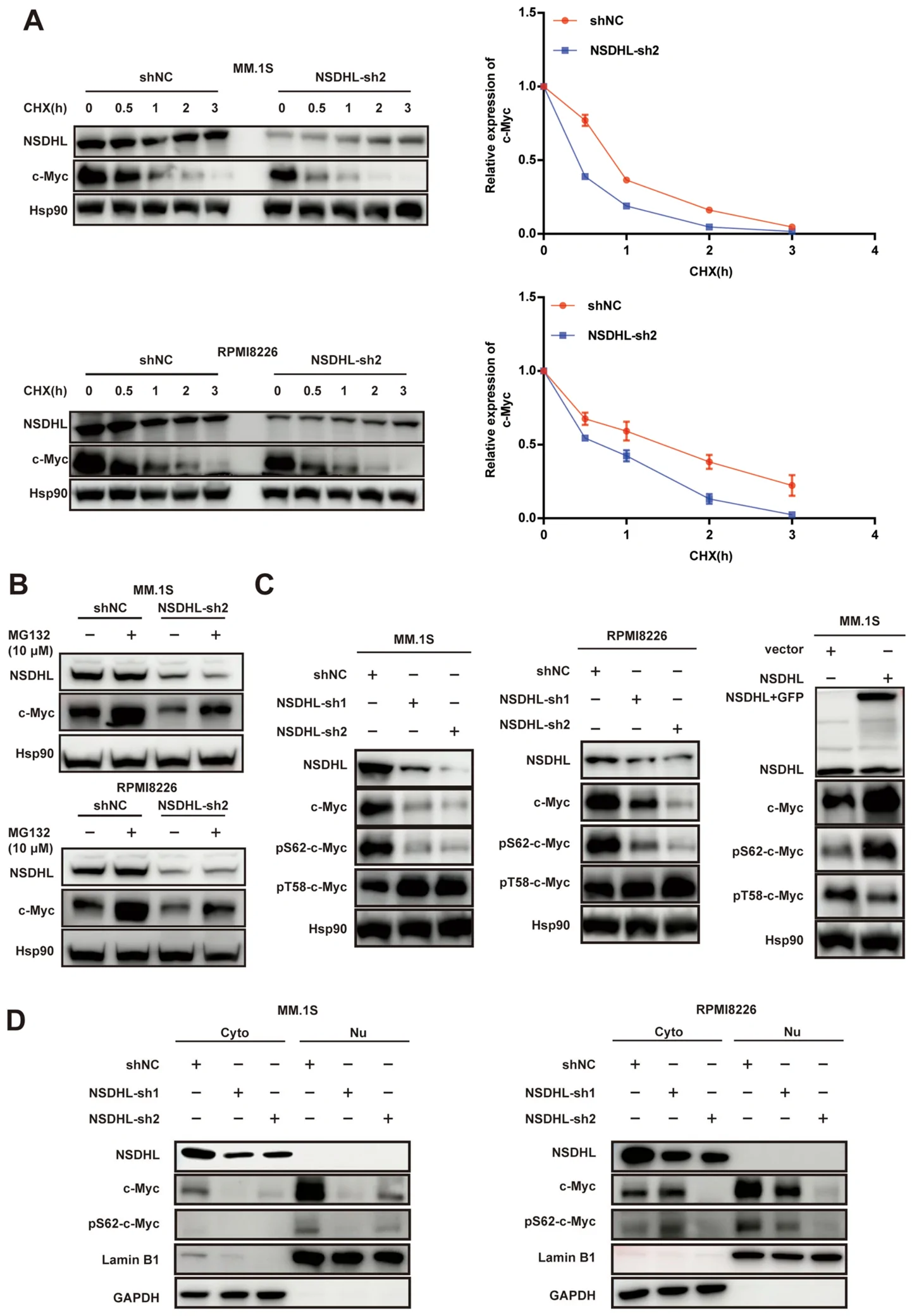
NSDHL stabilizes c-Myc protein in multiple myeloma cells. (**A**) CHX (20 μg/mL) chase assay of c-Myc protein stability in MM cells with NSDHL knockdown. c-Myc levels were analyzed by Western blot (left) and quantified by densitometry after normalization to Hsp90 (right). (**B**) c-Myc expression was evaluated by Western blot in MM cells with NSDHL knockdown following treatment with DMSO or proteasome inhibitor MG132 (10 μM, 6–8 h). (**C**) Western blot analysis of c-Myc, pS62-c-Myc, and pT58-c-Myc in MM cells with NSDHL knockdown or overexpression. (**D**) Western blot analysis of c-Myc expression in cytoplasmic and nuclear fractions of MM cells with NSDHL knockdown. GAPDH and Lamin B1 were used as internal controls for the cytoplasmic and nuclear fractions, respectively.

c-Myc is a short-lived protein whose stability can be enhanced by post-translational regulatory mechanisms, whereas its abundance is tightly controlled by ubiquitin–proteasome-mediated degradation [14]. To determine whether NSDHL regulates c-Myc protein stability, we examined c-Myc turnover by blocking protein synthesis with CHX or inhibiting proteasomal degradation with MG132. Compared with shNC cells, NSDHL-knockdown cells showed a more rapid decline in c-Myc protein levels following CHX treatment, indicating that NSDHL depletion shortened the half-life of c-Myc in MM cells (Figure 5A). Moreover, MG132 treatment markedly restored c-Myc protein expression reduced by NSDHL knockdown (Figure 5B), suggesting that NSDHL maintains c-Myc abundance, at least in part, by suppressing proteasome-dependent degradation.

Phosphorylation at Thr58 and Ser62 is essential for controlling c-Myc stability through the ubiquitin–proteasome pathway [14,15]. We therefore investigated whether NSDHL regulates these phosphorylation events. NSDHL knockdown decreased pS62-c-Myc levels but increased pT58-c-Myc levels, whereas NSDHL overexpression produced the opposite effects (Figure 5C). Furthermore, NSDHL depletion markedly reduced the nuclear accumulation of both c-Myc and pS62-c-Myc in MM cells (Figure 5D). Collectively, these findings indicate that NSDHL stabilizes c-Myc by modulating the Ser62/Thr58 phosphorylation balance and restraining proteasome-dependent degradation, thereby sustaining nuclear c-Myc accumulation in MM cells.

### 2.6. NSDHL Promoted Glycolysis and Cell Growth Through Stabilization of c-Myc

**Figure 6:**
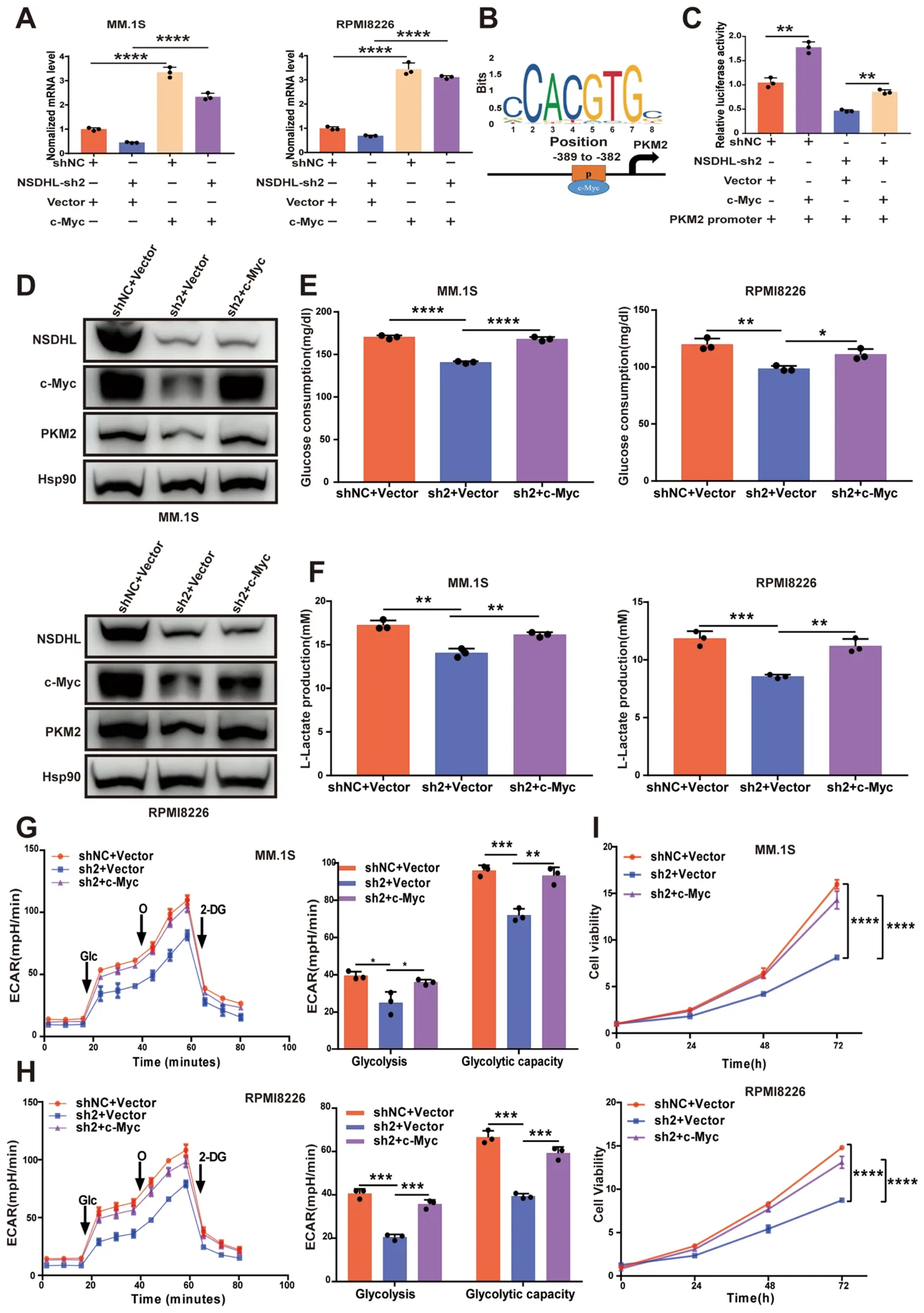
c-Myc mediated NSDHL-dependent regulation of PKM2, glycolysis, and proliferation in MM cells. (**A**) qRT-PCR analysis of PKM2 mRNA expression in NSDHL-knockdown MM.1S and RPMI8226 cells following transfection with vector or c-Myc plasmid. (**B**) JASPAR database-based prediction of potential c-Myc-binding motifs within the PKM2 promoter region. (**C**) Dual-luciferase reporter analysis of PKM2 promoter activity under the indicated conditions. (**D**) Western blot analysis of PKM2 protein expression in the indicated cells. (**E**,**F**) Glucose consumption (**E**) and lactate production (**F**) were assessed under the indicated conditions. (**G**,**H**) ECAR, glycolysis, and glycolytic capacity were assessed in the indicated cells under the indicated conditions. (**I**) CCK-8 analysis was performed to assess cell proliferation under the indicated conditions. Data are presented as mean ± SEM. * *p* < 0.05, ** *p* < 0.01, *** *p* < 0.001, **** *p* < 0.0001.

We next investigated whether c-Myc mediates the downstream effects of NSDHL on glycolytic reprogramming in MM cells by focusing on PKM2, a key glycolytic enzyme that catalyzes the final step of glycolysis and supports the Warburg phenotype in cancer cells [16,17,18,19,20]. It has been reported that c-Myc directly binds to the PKM2 promoter and promotes PKM2 transcription [21,22]. Therefore, we first examined whether ectopic c-Myc expression could rescue the reduction in PKM2 transcription caused by NSDHL depletion. NSDHL knockdown markedly decreased PKM2 mRNA levels in both MM.1S and RPMI8226 cells, whereas c-Myc overexpression largely reversed this effect (Figure 6A). Bioinformatic analysis identified a putative c-Myc-binding motif within the PKM2 promoter region (Figure 6B). Consistently, in 293T cells, c-Myc overexpression markedly activated PKM2 promoter-driven luciferase activity, whereas NSDHL knockdown significantly weakened the ability (Figure 6C). Western blot analysis showed ectopic c-Myc expression rescued PKM2 protein levels in NSDHL-deficient MM cells (Figure 6D). These results suggest that NSDHL regulates PKM2 expression, through c-Myc-dependent transcriptional activation.

In parallel, ectopic c-Myc expression markedly restored glucose consumption and lactate production in NSDHL-knockdown cells (Figure 6E,F). Consistent with these findings, extracellular acidification rate analysis showed that c-Myc re-expression substantially rescued the glycolytic defects caused by NSDHL depletion (Figure 6G,H). Functionally, NSDHL knockdown markedly suppressed MM cell viability over time, whereas restoration of c-Myc significantly attenuated this inhibitory effect (Figure 6I). Collectively, these findings indicate that NSDHL promotes PKM2 expression, glycolytic metabolism, and MM cell growth largely by sustaining c-Myc expression and activity.

## 3. Discussion

Metabolic reprogramming is a fundamental hallmark of malignant tumors, among which aerobic glycolysis, also known as the Warburg effect, is one of the most widely recognized metabolic alterations [23,24]. Accumulating evidence has shown that aberrant glycolytic metabolism is closely associated with MM cell proliferation, disease progression, and therapeutic response [25,26,27,28]. Thus, elucidating the regulatory mechanisms underlying aberrant glycolysis in MM may help identify actionable metabolic vulnerabilities and provide new opportunities for therapeutic intervention.

In the current study, we demonstrate that NSDHL plays an important role in MM progression and glycolytic metabolic reprogramming. Cancer cells preferentially rely on enhanced glycolysis, converting glucose to lactate even under oxygen-replete conditions, to support rapid tumor growth. Multiple mechanisms cooperate to promote enhanced glycolysis in cancer cells, with most of these mechanisms involving the upregulation of key glycolytic enzymes [6]. In line with this metabolic feature, we showed that NSDHL positively regulated the glycolytic enzymes HK2 and PKM2 in MM cells, and its expression correlated with HK2 and PKM2 levels in MM patient samples. These findings uncovered a previously unrecognized link between NSDHL and glycolytic reprogramming in MM cells.

Although NSDHL is a key enzyme involved in cholesterol biosynthesis, how it participates in glycolytic reprogramming in MM cells remains unclear. Our transcriptomic analysis provided an important clue to this question, as GSEA showed that MYC target gene signatures were significantly suppressed following NSDHL knockdown. MYC (c-Myc) is a tightly regulated proto-oncogene that integrates growth-promoting signals under physiological conditions, whereas its aberrant activation is frequently observed in human cancers [29]. In MM, MYC dysregulation is a key secondary oncogenic event in the progression from MGUS/SMM to aMM, and elevated c-Myc expression or MYC rearrangements are associated with aggressive disease features and poor clinical outcomes [3,30,31,32,33]. In our study, we show that NSDHL sustains high MYC protein levels in MM cells through the stabilization of MYC.

Previous studies have shown that c-Myc stability is dynamically regulated by sequential phosphorylation at Ser62 and Thr58. In response to growth-promoting signals, Ser62 phosphorylation can be induced by multiple kinases, including ERK, CDKs, JNK, PLK1, and PIM, leading to transient c-Myc stabilization and enhanced transcriptional activity. This event subsequently permits GSK-3β-mediated Thr58 phosphorylation, Ser62 dephosphorylation, and FBW7-dependent ubiquitination, thereby promoting proteasomal degradation of c-Myc [14,15,34,35]. Dysregulation of this phosphorylation-dependent turnover mechanism in cancer can lead to sustained c-Myc stabilization and oncogenic activity. Consistent with this model, we found that NSDHL knockdown decreased pS62-c-Myc but increased pT58-c-Myc, while NSDHL overexpression induced the opposite pattern. In addition, NSDHL depletion reduced nuclear accumulation of c-Myc and pS62-c-Myc, suggesting that NSDHL sustains both the stability and nuclear presence of functionally active c-Myc. These results provide a mechanistic explanation for how NSDHL enhances c-Myc-driven transcriptional programs in MM cells. However, the upstream signaling events linking NSDHL to c-Myc phosphorylation remain to be clarified. Future studies should determine whether NSDHL regulates kinases, phosphatases, or ubiquitin ligases involved in c-Myc turnover, such as ERK, GSK-3β, PP2A, or FBW7.

**Figure 7:**
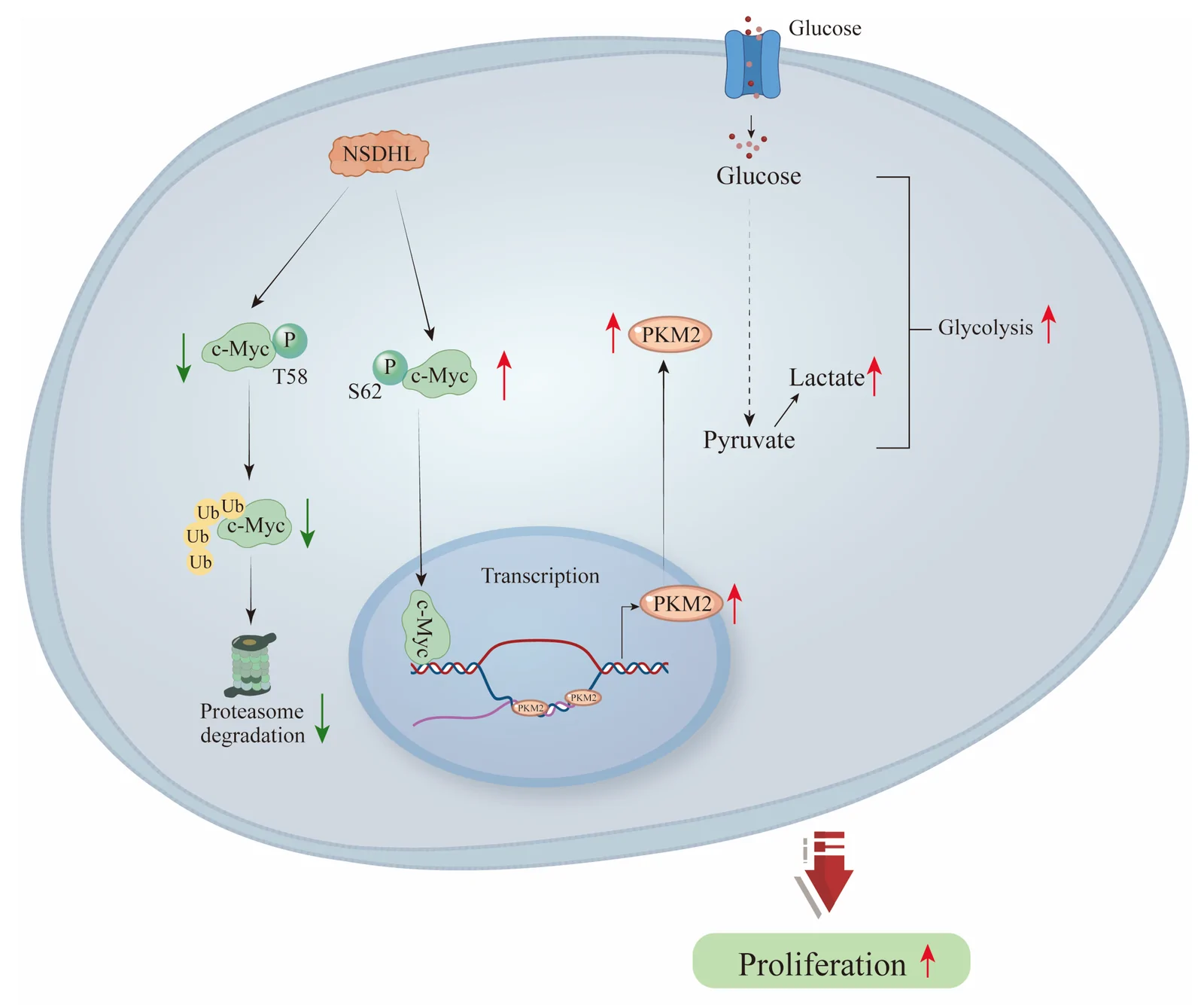
Schematic model of the NSDHL/c-Myc/PKM2 axis in MM glycolysis.

Importantly, ectopic c-Myc expression restored PKM2 mRNA and protein levels in NSDHL-knockdown cells and rescued glycolytic defects and growth inhibition. These data support a model in which NSDHL stabilizes c-Myc, thereby enhancing PKM2 expression, glycolytic activity, and MM cell proliferation (Figure 7). To our knowledge, the involvement of the NSDHL/c-Myc/PKM2 axis in glycolytic reprogramming of MM has not previously been characterized. Because direct pharmacological targeting of c-Myc remains challenging [36,37], disrupting upstream regulators such as NSDHL may represent an alternative strategy to indirectly suppress c-Myc-driven glycolysis and MM progression.

## 4. Materials and Methods

### 4.1. Bioinformatic Analysis

Transcriptomic profiles and clinical annotations for the MMRF-CoMMpass cohort were obtained from UCSC Xena. Additional MM-related datasets (GSE46816, GSE116294, GSE136337, GSE26760) were downloaded from GEO for independent validation. MMRF-CoMMpass STAR-Counts were normalized to TPM; GEO Affymetrix probes were mapped to gene symbols (highest-expressing probe retained per gene), and non-logged data were log2-transformed. GSE46816 (8 paired CD138^+^ and CD138^−^ cell fractions isolated from MM cell lines, with CD138^−^ representing the stem-like population) was used to compare NSDHL between fractions by paired *t*-test; GSE116294 compared normal donor vs MM plasma cells by pairwise *t*-tests; NSDHL across ISS stages was evaluated by one-way ANOVA with Tukey’s HSD. For survival, patients were stratified by median NSDHL (cut-offs: 7.08 TPM in MMRF-CoMMpass, *n* = 763; 5.99 log2-transformed in GSE136337, *n* = 426), with KM curves compared by log-rank test. Significance: *p* < 0.05. All analyses used R 4.4.3 with packages TCGAbiolinks/GEOquery (retrieval), DESeq2 (differential expression), clusterProfiler (GO), survival/survminer (survival), and ggplot2 (visualization).

### 4.2. Cell Culture

The human MM cell line MM.1S, RPMI 8266 and HEK293T were purchased from Procell Life Science& Technology Co., Ltd. (Wuhan, China), with the following catalog numbers: MM.1S (CL-0614), RPMI8226 (CL-0564), and HEK-293T (CL-0005). MM.1S and RPMI8226 cells were cultured in RPMI-1640 medium (GIBCO, 11875093) plus 10% FBS (Corning, 10091148). HEK293T cells were maintained in DMEM (GIBCO, 11965092) plus 10% FBS. All cells were cultured at 37 °C in a humidified incubator containing 5% CO_2_.

### 4.3. Lentiviral Transduction and Plasmid Transfection

Stable NSDHL-knockdown and NSDHL-overexpressing MM cell lines were generated using lentiviral vectors purchased from Ruibiotech (Guangzhou, China). Two shRNAs targeting human NSDHL were cloned into the pLKO.1-puro vector: shNSDHL-1, 5’-TCCTGACAGGCCTCAATTATG-3’, and shNSDHL-2, 5’-CCAACGATCCTGAGAAGAATT-3’. Full-length human NSDHL was cloned into the psin-ef1-puro vector for overexpression. MM.1S and RPMI8226 cells were infected with the indicated lentiviruses at an MOI of 20 in the presence of 10 μg/mL polybrene. After 10–24 h, the medium was replaced with fresh complete RPMI-1640 medium, followed by selection with 2 μg/mL puromycin 48 h later. Stable cell lines were validated by qRT-PCR and Western blotting before subsequent experiments. For rescue assays, cells were transiently transfected with a c-Myc overexpression plasmid or empty vector using Lipo3000 (Invitrogen, L3000015, Carlsbad, CA, USA).

### 4.4. RNA Isolation and RT-qPCR

Total RNA was isolated from cells using TRIzol reagent (Invitrogen, 15596018) following the manufacturer’s protocol. Subsequently, first-strand cDNA was synthesized using the RevertAid First Strand cDNA Synthesis Kit (Thermo Scientific, K1622, Waltham, MA, USA). Quantitative real-time PCR was conducted using TB Green Premix Ex Taq reagent (TakaraRR420A, Kusatsu, Japan). β-actin served as the endogenous reference gene, and relative gene expression was analyzed using the 2^^−ΔΔCt^ method. All reactions were performed in triplicate. The primer sequences are provided in Table 1.

### 4.5. Western Blotting and Nuclear/Cytoplasmic Fractionation

Total protein was extracted using RIPA lysis buffer containing 50 mM Tris-HCl, 150 mM NaCl, 5 mM EDTA, and 0.5% Nonidet P-40, supplemented with 1% protease and phosphatase inhibitors (Thermo Scientific, 78443). For nuclear and cytoplasmic fractionation, cells were collected, washed with cold PBS, and processed using a Nuclear and Cytoplasmic Protein Extraction Kit (Beyotime, Shanghai, China) according to the manufacturer’s instructions. Protein concentrations were determined using a BCA protein assay kit (Beyotime, China). Equal amounts of protein were separated by SDS-PAGE and transferred onto 0.45 μm PVDF membranes (Millipore, IPVH00010, Billerica, MA, USA). After blocking with 5% non-fat milk (BD Difco, 232100, Franklin Lakes, NJ, USA), the membranes were incubated overnight at 4 °C with primary antibodies against NSDHL (Proteintech, Tokyo, Janpan, 15111-1-AP), HK2 (Proteintech, 66974-1-Ig), PKM2 (Proteintech, 60268-1-Ig), c-Myc (Abcam, ab32072, Cambridge, UK), phospho-c-Myc Ser62 (Abcam, ab185656), phospho-c-Myc Thr58 (Abcam, ab185655), Lamin B1 (Proteintech, 12987-1-AP), HSP90 (Proteintech, 13171-1-AP), GAPDH (Proteintech, 60004-1-Ig) or α-tubulin (Proteintech, 66031-1-Ig). After incubation with HRP-conjugated secondary antibodies, protein bands were visualized using enhanced chemiluminescence reagents. Lamin B1 was used as the nuclear marker, whereas GAPDH, HSP90 or α-tubulin was used as the cytoplasmic/loading control.

### 4.6. Cell Viability Assay

Cell proliferation was assessed using the Cell Counting Kit-8 assay (Biosharp, BS350B, Hefei, China). Briefly, MM cells were seeded into 96-well plates (8 × 10^3^/well). At the indicated time points, CCK-8 reagent was added to each well and incubated at 37 °C for 1 h. Absorbance at 450 nm was measured using a microplate reader.

### 4.7. Measurement of Glucose Consumption and Lactate Production

Cells were seeded at equal densities and cultured under the indicated experimental conditions. Culture supernatants were collected after incubation, and glucose and lactate levels were measured using Glucose Assay Kit with O-toluidine (Beyotime, China) and L-Lactate Assay Kit with WST-8 (Beyotime, China) according to the manufacturers’ protocols.

Glucose concentration was determined at 630 nm based on the standard curve, and glucose consumption was calculated as the difference between glucose levels in control medium and cell-conditioned medium. Lactate concentration was measured at 450 nm according to the standard curve, and lactate production was calculated after subtracting the blank value.

### 4.8. Extracellular Acidification Rate Analysis (ECAR)

ECAR was measured using a Seahorse XF96 Extracellular Flux Analyzer with a Seahorse XF Glycolysis Stress Test Kit according to the manufacturer’s instructions (Agilent Technologies, Santa Clara, CA, USA). Briefly, MM cells (5 × 10^4^ per well) were seeded onto Seahorse XF96 cell culture microplates and equilibrated in assay medium before detection. Glucose (10 mM), oligomycin (1 μM), and 2-DG (50 mM) were sequentially injected according to the manufacturer’s protocol. Basal glycolysis and glycolytic capacity were calculated based on ECAR changes and normalized to protein concentration.

### 4.9. RNA Sequencing and Enrichment Analysis

Total RNA was extracted from MM.1S cells stably transduced with shNC or NSDHL-sh1 (three biological replicates per group) using TRIzol (Invitrogen). RNA-seq libraries were prepared using the NEBNext Ultra II RNA Library Prep Kit (Ipswich, MA, USA) and subjected to 150 bp paired-end sequencing on the NovaSeq 6000 platform (Illumina, San Diego, CA, USA) at Shanghai Biotree Biotech Co., Ltd (Shanghai, China). Raw reads were processed by fastp (v0.23.4) for adapter trimming and quality filtering (Q20 sliding-window), and the resulting clean reads were aligned to the GRCh38/hg38 reference genome employing HISAT2 (v2.2.1). Gene-level abundance was estimated by StringTie2 (v2.1.4) and normalized as FPKM and TPM. Differential expression analysis between NSDHL-sh1 and shNC groups was carried out with DESeq2 (v1.38.3), and genes with |log2FoldChange| > 1 and padj < 0.05 were considered differentially expressed. GO and KEGG enrichment analyses were conducted using clusterProfiler (v4.6.2) with padj < 0.05 as the significance threshold. GSEA was performed using the same package, ranking all genes by log2FoldChange against hallmark gene sets from MSigDB (v7.2), with significance set at padj < 0.05. The RNA-seq data generated in this study have been deposited in the NCBI Sequence Read Archive (SRA) under BioProject accession number PRJNA1525188.

### 4.10. Immunofluorescence Staining

Cells were fixed with 4% paraformaldehyde, permeabilized with Triton X-100(0.05%), and blocked with BSA (2%). Cells were then incubated with primary antibodies against c-Myc (Proteintech, 10828-1-AP, 1:200) overnight at 4 °C, followed by incubation with fluorescence-conjugated secondary antibodies (EnkiLife, Wuhan, China). Nuclei were counterstained with 4,6-diamidino-2-phenylindole (DAPI, Invitrogen, 62248). Fluorescence images were captured using a fluorescence or confocal microscope (Cell-Xtreme, Guangzhou, China).

### 4.11. Dual-Luciferase Reporter Assay

A fragment of the human PKM promoter was cloned into the pGL3-Basic luciferase reporter vector. Primers listed as below:

pGL3-PKM-F: TTTCTCTATCGATAGGTACCGCTGGCACCAGGCCTCCAAGCTTGATCTTC;

pGL3-PKM-R: AGTACCGGAATGCCAAGCTTGGCCTACCTGGGCAGGGCCCAG;

HEK293T cells were seeded into 24-well plates and co-transfected with the PKM promoter reporter plasmid, pRL-TK Renilla luciferase plasmid, c-Myc expression vector or empty vector, and NSDHL-shRNA or control shRNA plasmid as indicated. After transfection, cells were lysed, and luciferase activity was measured using a Dual-Luciferase Reporter Assay System (Promega, E1960) according to the manufacturer’s protocol. Firefly and Renilla luciferase luminescence signals were detected using a luminescence-compatible microplate reader (Synergy H1, Winooski, VT, USA). Firefly luciferase activity was normalized to Renilla luciferase activity, and relative promoter activity was expressed as fold change relative to the corresponding control group.

### 4.12. Statistical Analysis

All experiments were performed at least three independent times unless otherwise stated. Data are presented as the mean ± SEM. Statistical analyses were performed using GraphPad Prism 8.0 software. Differences between two groups were analyzed using two-tailed Student’s *t*-test, whereas comparisons among multiple groups were performed using one-way ANOVA. * *p* < 0.05, ** *p* < 0.01, *** *p* < 0.001.

## Figures and Tables

**Table 1:** Primer sequences for RT–qPCR.

Gene	Primer Sequences (5’-3’)
β-actin	F: AGCGAGCATCCCCCAAAGTT
	R: GGGCACGAAGGCTCATCATT
*NSDHL*	F: AAGAGATGCACAGTGATCGGT
	R: GCACCTGGGGATTATCAAACC
*PKM2*	F: CGTCATTCATCCGCAAGGCAT
	R: CACGAGCCACCATGATCCCA
*HK2*	F: GTGAATCGGAGAGGTCCCAC
	R: GCTAACTTCGGCCACAGGAT
*c-Myc*	F: GTCAAGAGGCGAACACACAAC
	R: TTGGACGGACAGGATGTATGC

## Data Availability

The raw RNA-seq data generated in this study are available in the NCBI SRA under BioProject PRJNA1525188.

## References

[1] Bray F., Laversanne M., Sung H., Ferlay J., Siegel R.L., Soerjomataram I., Jemal A. (2024). Global cancer statistics 2022: GLOBOCAN estimates of incidence and mortality worldwide for 36 cancers in 185 countries. CA Cancer J. Clin..

[2] Mafra A., Laversanne M., Marcos-Gragera R., Chaves H.V.S., Mcshane C., Bray F., Znaor A. (2025). The global multiple myeloma incidence and mortality burden in 2022 and predictions for 2045. JNCI J. Natl. Cancer Inst..

[3] Malard F., Neri P., Bahlis N.J., Terpos E., Moukalled N., Hungria V.T.M., Manier S., Mohty M. (2024). Multiple myeloma. Nat. Rev. Dis. Primers.

[4] Morris J.L.M., Webster R.M. (2025). The multiple myeloma drug market. Nat. Rev. Drug Discov..

[5] Pavlova N.N., Zhu J.J., Thompson C.B. (2022). The hallmarks of cancer metabolism: Still emerging. Cell Metab..

[6] Hanahan D., Weinberg R.A. (2011). Hallmarks of Cancer: The Next Generation. Cell.

[7] Purcell E., Dowling P., O’Gorman P., Bazou D. (2025). Metabolic alterations in multiple myeloma and extramedullary disease- a novel niche of therapeutic targets. Blood Rev..

[8] Kim D.-G., Cho S., Lee K.-Y., Cheon S.-H., Yoon H.-J., Lee J.-Y., Kim D., Shin K.-S., Koh C.-H., Koo J.S. (2020). Crystal structures of human NSDHL and development of its novel inhibitor with the potential to suppress EGFR activity. Cell. Mol. Life Sci..

[9] Chen M., Zhao Y., Yang X., Zhao Y., Liu Q., Liu Y., Hou Y., Sun H., Jin W. (2021). NSDHL promotes triple-negative breast cancer metastasis through the TGFβ signaling pathway and cholesterol biosynthesis. Breast Cancer Res. Treat..

[10] Yoon S.-H., Lee S., Kim H.S., Song J., Baek M., Ryu S., Lee H.-B., Moon H.-G., Noh D.-Y., Jon S. (2024). NSDHL contributes to breast cancer stem-like cell maintenance and tumor-initiating capacity through TGF-β/Smad signaling pathway in MCF-7 tumor spheroid. BMC Cancer.

[11] Xiao Y., Xie J., Liu L., Huang W., Han Q., Qin J., Liu S., Jiang Z. (2020). NAD(P)-dependent steroid dehydrogenase-like protein and neutral cholesterol ester hydrolase 1 serve as novel markers for early detection of gastric cancer identified using quantitative proteomics. J. Clin. Lab. Anal..

[12] Sukhanova A., Gorin A., Serebriiskii I.G., Gabitova L., Zheng H., Restifo D., Egleston B.L., Cunningham D., Bagnyukova T., Liu H. (2013). Targeting C4-Demethylating Genes in the Cholesterol Pathway Sensitizes Cancer Cells to EGF Receptor Inhibitors via Increased EGF Receptor Degradation. Cancer Discov..

[13] Huang H.-Y., Wang Y., Wang W.-D., Wei X.-L., Gale R.P., Li J.-Y., Zhang Q.-Y., Shu L.-L., Li L., Li J. (2021). A prognostic survival model based on metabolism-related gene expression in plasma cell myeloma. Leukemia.

[14] Hann S.R. (2006). Role of post-translational modifications in regulating c-Myc proteolysis, transcriptional activity and biological function. Semin. Cancer Biol..

[15] Junttila M.R., Westermarck J. (2008). Mechanisms of MYC stabilization in human malignancies. Cell Cycle.

[16] Israelsen W.J., Vander Heiden M.G. (2015). Pyruvate kinase: Function, regulation and role in cancer. Semin Cell Dev. Biol..

[17] Zhang Z., Deng X., Liu Y., Liu Y., Sun L., Chen F. (2019). PKM2, function and expression and regulation. Cell Biosci..

[18] Christofk H.R., Vander Heiden M.G., Harris M.H., Ramanathan A., Gerszten R.E., Wei R., Fleming M.D., Schreiber S.L., Cantley L.C. (2008). The M2 splice isoform of pyruvate kinase is important for cancer metabolism and tumour growth. Nature.

[19] Liu L., Wang J., Liang L., Chen C., Xie M., Tang W. (2025). Targeting PKM2 in cancer therapeutics: Mechanistic advances and translational opportunities. Front. Immunol..

[20] Zulfareen, Taiyab A., Hasan G.M., Hassan M.I. (2025). A Review on the Role of Pyruvate Kinase M2 in Cancer: From Metabolic Switch to Transcriptional Regulation. Int. J. Biol. Macromol..

[21] Ma J., Sun F., Li W., Du R., Liu M., Wei Q., Kang B., Yan S., Wang C. (2024). SULT2B1: A novel therapeutic target in colorectal cancer via modulation of AKT/PKM2-mediated glycolysis and proliferation. J. Transl. Med..

[22] Zhao R., Yi Y., Liu H., Xu J., Chen S., Wu D., Wang L., Li F. (2024). RHOF promotes Snail1 lactylation by enhancing PKM2-mediated glycolysis to induce pancreatic cancer cell endothelial–mesenchymal transition. Cancer Metab..

[23] Heiden M.G.V., Cantley L.C., Thompson C.B. (2009). Understanding the Warburg Effect: The Metabolic Requirements of Cell Proliferation. Science.

[24] Koppenol W.H., Bounds P.L., Dang C.V. (2011). Otto Warburg’s contributions to current concepts of cancer metabolism. Nat. Rev. Cancer.

[25] Gu Z., Xia J., Xu H., Frech I., Tricot G., Zhan F. (2017). NEK2 Promotes Aerobic Glycolysis in Multiple Myeloma Through Regulating Splicing of Pyruvate Kinase. J. Hematol. Oncol..

[26] Jia M., Fu Z., Ye C., Xu W., Liu J., Wu C., Yan H. (2025). Targeting MTHFD2 alters metabolic homeostasis and synergizes with bortezomib to inhibit multiple myeloma. Cell Death Discov..

[27] Wu Y., Luo Y., Yao X., Shi X., Xu Z., Re J., Shi M., Li M., Liu J., He Y. (2024). KIAA1429 increases FOXM1 expression through YTHDF1–mediated m6A modification to promote aerobic glycolysis and tumorigenesis in multiple myeloma. Cell Biol. Toxicol..

[28] Xue W., Li Y., Ma Y., Zhang F. (2025). GDF15-mediated enhancement of the Warburg effect sustains multiple myeloma growth via TGFβ signaling pathway. Cancer Metab..

[29] Dang C.V. (2012). MYC on the Path to Cancer. Cell.

[30] Cristóbal-Vargas S., Cuadrado M., Gutiérrez N.C. (2025). MYC alterations in multiple myeloma: Genetic insights and prognostic impact. Neoplasia.

[31] Misund K., Network M.C., Keane N., Stein C.K., Asmann Y.W., Day G., Welsh S., Van Wier S.A., Riggs D.L., Ahmann G. (2020). MYC dysregulation in the progression of multiple myeloma. Leukemia.

[32] Avet-Loiseau H., Gerson F., Magrangeas F., Minvielle S., Harousseau J.-L., Bataille R. (2001). Rearrangements of the c-myc oncogene are present in 15% of primary human multiple myeloma tumors. Blood.

[33] Walker B.A., Wardell C.P., Brioli A., Boyle E., Kaiser M.F., Begum D.B., Dahir N.B., Johnson D.C., Ross F.M., Davies F.E. (2014). Translocations at 8q24 juxtapose with genes that harbor superenhancers resulting in overexpression and poor prognosis in myeloma patients. Blood Cancer J..

[34] Boi D., Rubini E., Breccia S., Guarguaglini G., Paiardini A. (2023). When Just One Phosphate Is One Too Many: The Multifaceted Interplay between Myc and Kinases. Int. J. Mol. Sci..

[35] Farrell A.S., Sears R.C. (2014). MYC Degradation. Cold Spring Harb. Perspect. Med..

[36] Jovanović K.K., Roche-Lestienne C., Ghobrial I.M., Facon T., Quesnel B., Manier S. (2018). Targeting MYC in multiple myeloma. Leukemia.

[37] Wang C., Fang H., Zhang J.W., Gu Y. (2021). Targeting “undruggable” c-Myc protein by synthetic lethality. Front. Med..

